# An intelligent detection method for plasmodium based on self-supervised learning and attention mechanism

**DOI:** 10.3389/fmed.2023.1117192

**Published:** 2023-06-29

**Authors:** Min Fu, Kai Wu, Yuxuan Li, Linkai Luo, Wei Huang, Qi Zhang

**Affiliations:** ^1^School of Aerospace Engineering, Xiamen University, Xiamen, China; ^2^Department of Schistosomiasis and Endemic Diseases, Wuhan Center for Disease Control and Prevention, Wuhan, China; ^3^School of Art and Design, Wuhan University of Technology, Wuhan, China; ^4^Department of Orthopaedics, Union Hospital, Tongji Medical College, Huazhong University of Science and Technology, Wuhan, China; ^5^Wuhan Jinyintan Hospital, Tongji Medical College of Huazhong University of Science and Technology, Wuhan, China

**Keywords:** deep learning, plasmodium parasites, self-supervised learning, attention mechanism, automatic detecting system

## Abstract

**Background:**

Malaria remains a severe life-threatening disease caused by plasmodium parasites. Microscopy is widely used for malaria diagnosis. However, it relies heavily on the skills and experience of inspectors. Due to low-level medical services and the lack of skilled inspectors, misdiagnoses are frequently made in some areas.

**Methods:**

In recent years, many successful applications of CNN models have been reported. Unlike images in the ImageNet, the image of plasmodium only has a tiny defect area with a large amount of information. In addition, the dataset is extremely unbalanced: the number of positive samples is much less than that of negative samples. This paper proposes a classification network by combining attention mechanism and ResNeSt for plasmodium detection and using self-supervised learning to pre-train the network. First, the positive samples were adopted to pre-train the network. Then, attention modules were taken to highlight the feature area. To support current and future research, we also constructed a plasmodium dataset with Plasmodium falciparum, Plasmodium vivax, Plasmodium ovale, and Plasmodium malaria and non-Plasmodium. Through self-supervised learning, a large amount of unlabeled data is used to mine the representational features, thus improving the feature extraction capability of the model and achieving higher accuracy, while saving the physician’s labeling time and improving the classification accuracy.

**Results:**

The experiments show that our model exhibits an excellent performance and that the test accuracy, sensitivity, and specificity attain 97.8%, 96.5%, and 98.9%, respectively.

**Conclusion:**

The AI classification method proposed in this paper can effectively assist clinicians in the diagnosis and provide a basis for the automatic detection of malaria parasites in the future.

## 1. Introduction

Malaria is a parasitosis transmitted by the bite of female anopheles. It is one of three major public health problems across the world. According to the WHO Malaria Report (1), the year 2020 witnessed 241 million malaria cases worldwide, including over 627,000 death cases. The vast majority of the cases were from Africa and Southeast Asia. Malaria was extensively distributed across Mainland China. Even at the beginning of this century, hundreds of thousands of malaria cases were discovered each year (2). With the efficacious control of malaria, the number of patients decreased year by year, and no local malaria cases had been reported by 2017 (3). The laboratory diagnosis of malaria mainly includes microscopy, rapid diagnostic tests for plasmodium antigen (RDT) and nucleic acid detection. Microscopic examination is the gold standard for malaria diagnosis thanks to its advantages of simple operation, rapidness, low cost, etc. However, the accuracy of microscopic examination depends heavily on the personal skills and experience of inspectors. With the decrease in malaria cases, microscopic examination has gradually become an inevitable trend in terms of detecting plasmodium. Over the past years, the incidence of imported malaria has gradually built up, and missed or wrong detections have been detected in microscopic examination (4). Moreover, microscopic examination requires the inspectors to have high-level skills and rich experience, leading to great differences in the detection results between different regions and different people. It is especially serious in areas with weak medical and health foundations.

There have been some works using convolutional neural networks to detect malaria parasites. Rajaraman et al. (5) evaluated the performance of deep learning-based pretrained CNN models as feature extractors in classifying infected and non-infected cells where the region of interest (ROI) was obtained by the Laplacian of Gaussian (LoG). They achieved a positive predictive value (PPV) of 94.44% with a sensitivity value of 96.20% which indicates the superiority of the pre-trained ResNet-50 (6). Peñas et al. (7) built a system that can detect the presence of malaria parasites and identify the type of parasite species in blood samples. In this study, a series of morphological transformations were implemented prior to the segmentation with connected components analysis and the classification with CNN. They showed an accuracy of 92.40% for parasite detection and 87.90% for the identification of parasite types. Liang et al. (6) designed a new CNN model with 17 layers for the classification of blood cells. The average classification accuracy of the CNN model was 97.37%, while the sensitivity, specificity and precision also reached the level of 97%. It suggested that the proposed CNN model is superior to the transfer learning model with pre-trained AlexNet. With the advent of convolutional neural networks (CNNs), better performance has been realized in image classification, object detection, attention prediction, and so on. The detection results with CNNs are not correlated with regions or inspectors. The algorithm is expected to diagnose plasmodium with CNNs. In our research, we introduced a CNN model with spatial attention modules and channel attention modules to diagnose plasmodium. Besides, data enhancement was employed to eliminate color influence. The experiments show that our algorithm achieves excellent performance.

## 2. Related works

Abhik Paul (7) developed three convolutional neural network (CNN) models for predicting the occurrence of malaria from images of red blood cells. Infected parasitic red blood cells and uninfected parasitic red blood cells. Finally, in the three settings, the proposed CNN setup-1, with a kernel size of 3 × 3 and a pool size of 2 × 2, achieved an accuracy of 96%.

Rahman et al. (8) transformed a malaria parasite object detection dataset into a classification dataset, making it the largest malaria classification dataset (63,645 cells), and evaluated the performance of several state-of-the-art deep neural network architectures pretrained on both natural and medical images on this new dataset. We provide deeper insights into the influence of synthetic images for the class imbalance problem in the malaria diagnosis context.

Madhu G et al. (9) developed an Imperative Dynamic routing mechanism with fully trained capsule networks for malaria classification. This model identifies the presence of malaria parasites by classifying thin blood smears containing samples of parasitized and healthy erythrocytes. Such proposed model has been evaluated and compared with novel machine vision models that evolved over a decade such as VGG, ResNet, DenseNet, and MobileNet. Through the assistance of the proposed capsule network, the problems in previous research have been well addressed.

The above studies reflect that AI performs well in detecting and classifying plasmodium images. Nevertheless, it only carries out classification on the cropped thin blood smear image that is not practical in the real world. The detection and classification of a single picture have no clinical significance. We extracted a whole pathological slice and then divided it into small pictures for detection so that we could diagnose whether a person has malaria instead of just offering a picture that displays diagnostic outcomes, which is of great help to assist clinicians in making judgments.

## 3. Methods

In this section, a plasmodium classification method predicated on ResNeSt (10) is put forward. Since malaria plasmodium appeared anywhere in the picture, we proposed two methods to improve our training accuracy, respectively, during self-supervised learning in pre-training and supervised learning in training:

1. We utilized the self-supervised method during the pre-training stage. First, we selected pictures without malaria plasmodium; then we obtained the cells’ location *via* segmentation; finally, we masked the cells and area at the same time instead of only masking the area during training for the purpose of enhancing training accuracy. Self-supervised learning by masking regions of cells allows the network to learn the features better.
2. Since malaria parasites showed up in any position. Also, Plasmodium staining is essentially the same, meaning that the features under certain color channels are the most distinct，we acquired better features through channel attention modules and spatial attention modules in the stage of supervised learning.

### 3.1. Datasets

The images of blood slices were retrospectively collected from the Department of Schistosomiasis and Endemic Diseases, Wuhan City Center between January 1, 2015 and February 29, 2020. They were labeled into two categories: confirmed plasmodium and non-plasmodium. The ages of all enrolled patients ranged from 18 to 75. Identifiable personal information, such as the names of patients, the names of hospitals, etc., was removed. The consecutive images of blood slices for each patient were selected for image recognition. A patient was classified as “plasmodium” as long as an image was identified with plasmodium. Otherwise, he was classified as “non-plasmodium.”

The images were labeled by three experts with more than 10 years of clinical experience. A total of 30,033 blood slice images with 100 patients were employed to train our model. These images were randomized to the training set and validation set at a ratio of 3:1. Another test set with 12,784 blood slice images was exploited to independently evaluate the performance of our model. Table 1 details the basic information of the datasets.

**Table 1:** The basic information of the datasets.

	Training and validation set	Test set
Plasmodium	19,976	8,897
Non-plasmodium	10,057	3,887

### 3.2. Image enhancement

The color difference that occurs in Pathological images often influences the performance of classification. We applied image enhancement in SimCLR (11) to scale the dataset and eliminate the influence of color. First, a random patch of images was selected and resized to 224 × 224 with a random horizontal flip, followed by a color distortion, consisting of a random sequence of brightness, contrast, saturation, hue adjustments, and an optional grayscale conversion. Then, Gaussian blur and solarization were applied to the patches.

### 3.3. Image embedding by self-supervised learning

In deep learning, it is a frequent problem that there is not sufficient labeled data. The size of our labeled dataset is still inadequate for the detection of plasmodium. Considering the high cost of manually labelling data, we adopted BYOL (12), a self-supervised learning approach, to alleviate the problem of insufficient labeled data. BYOL is a powerful self-supervised learning method and does not need any negative samples. It only requires that similar samples have similar representations.

**Figure 1:**
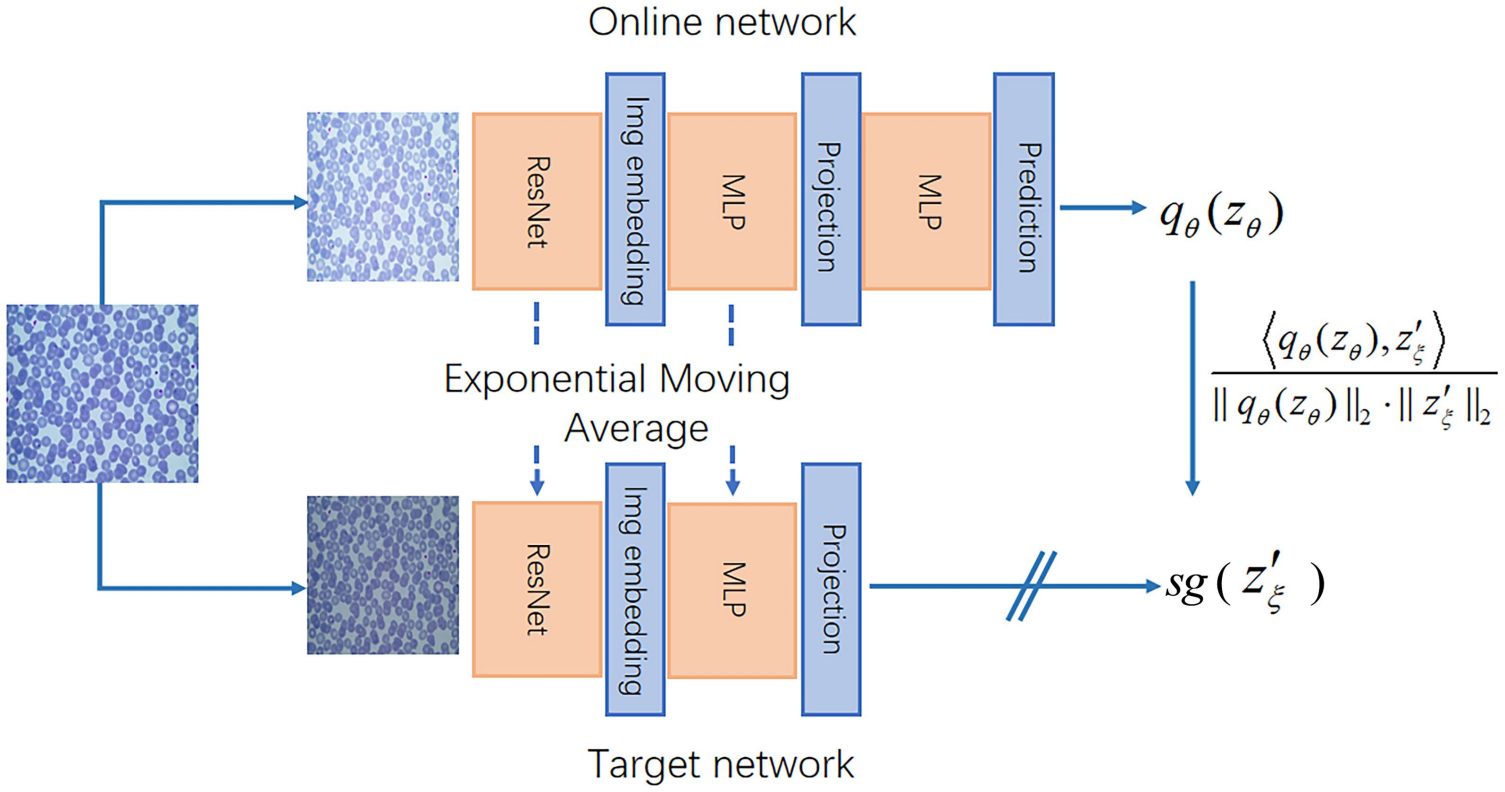
BYOL’s architecture. BYOL minimizes a similarity loss between $q_{\theta }$ ( $z_{\theta }$ ) and sg( $z_{\xi }$ ), where θ are the trained weights, ξ are an exponential moving average of θ and sg( $z_{\xi }$ ) means stop-gradient. At the end of training, everything but $f_{\theta }$ was discarded, and $y_{\theta }$ was used as the image representation.

An architecture of BYOL is detailed in Figure 1. There are two neural networks: an online network and a target network. The two networks share the same structure. The θ and ξ are the parameters of the online network and the target network, respectively. The θ was updated by minimizing a similarity loss between $q_{\theta }$ ( $z_{\theta }$ ) and sg( $z_{\xi }$ ) where sg means stop-gradient, while the ξ was updated by an exponential moving average of θ . After the training, the image embedding in the online network was utilized as the representation. BYOL has the advantages of high training efficiency and robustness to systematic deviation.

For malaria images, the cost of labelling is extremely high in that there are fewer doctors diagnosing malaria now. Thus, we only employed positive pictures for the self-supervised training of the model.

In the training, the images were rotated, color transformed, and subjected to different random cropping to get different positive sample pairs, and then input to the two networks. Since malaria parasites usually show up in cells, the pictures of malaria parasites are usually composed of cells. Therefore, when we masked the image, we first obtained the location of the cells through image segmentation. In the process of masking, we chose the masked cells, which is equivalent to improving the data set and distribution of positive samples with a view to promoting the efficiency of pre-training. We trained the online network to predict the target network representation of the same image under various enhanced views. Meanwhile, we used the slow moving average of the online network to update the target network. Furthermore, we kept the online network as our pre-training network subsequent to convergence.

### 3.4. Channel attention module and spatial attention module

**Figure 2:**
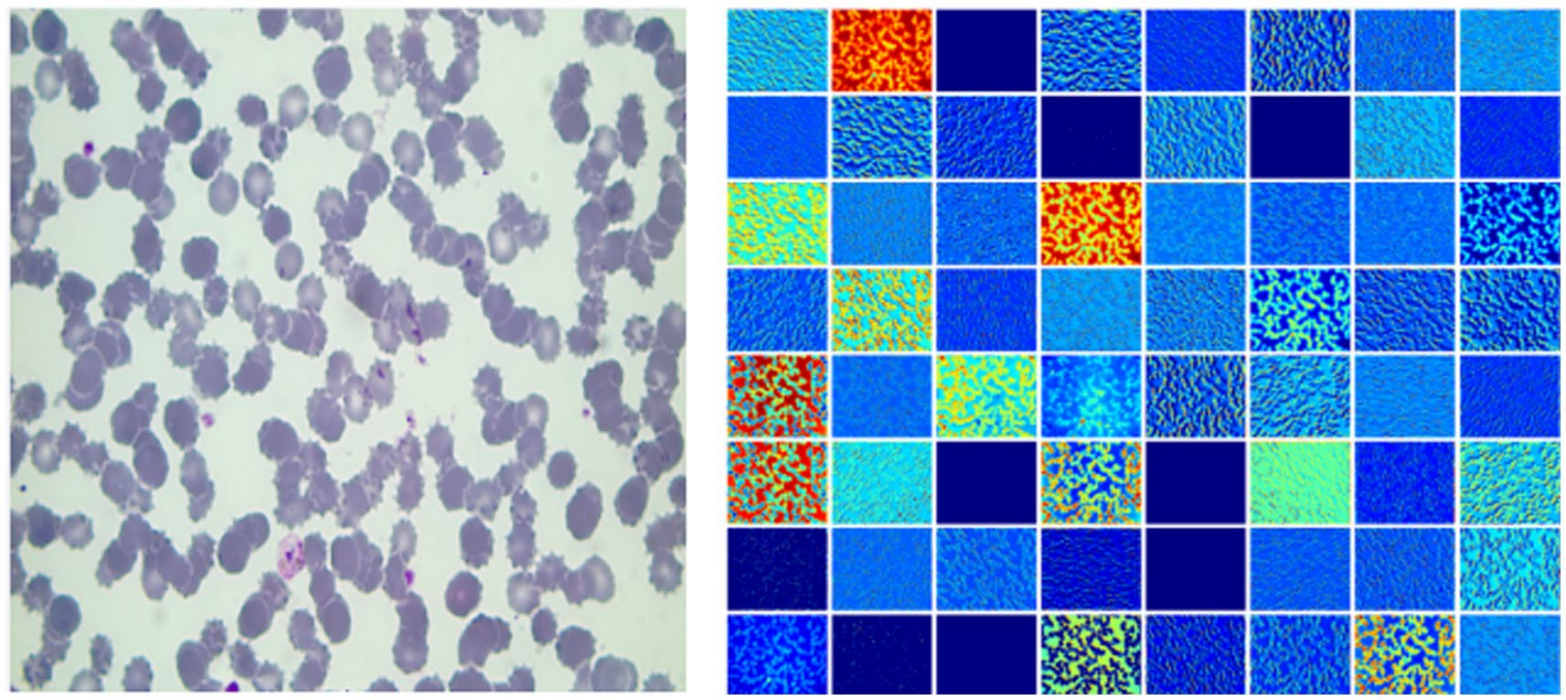
The left image is a plasmodium image, and the right image is the heat map.

**Figure 3:**
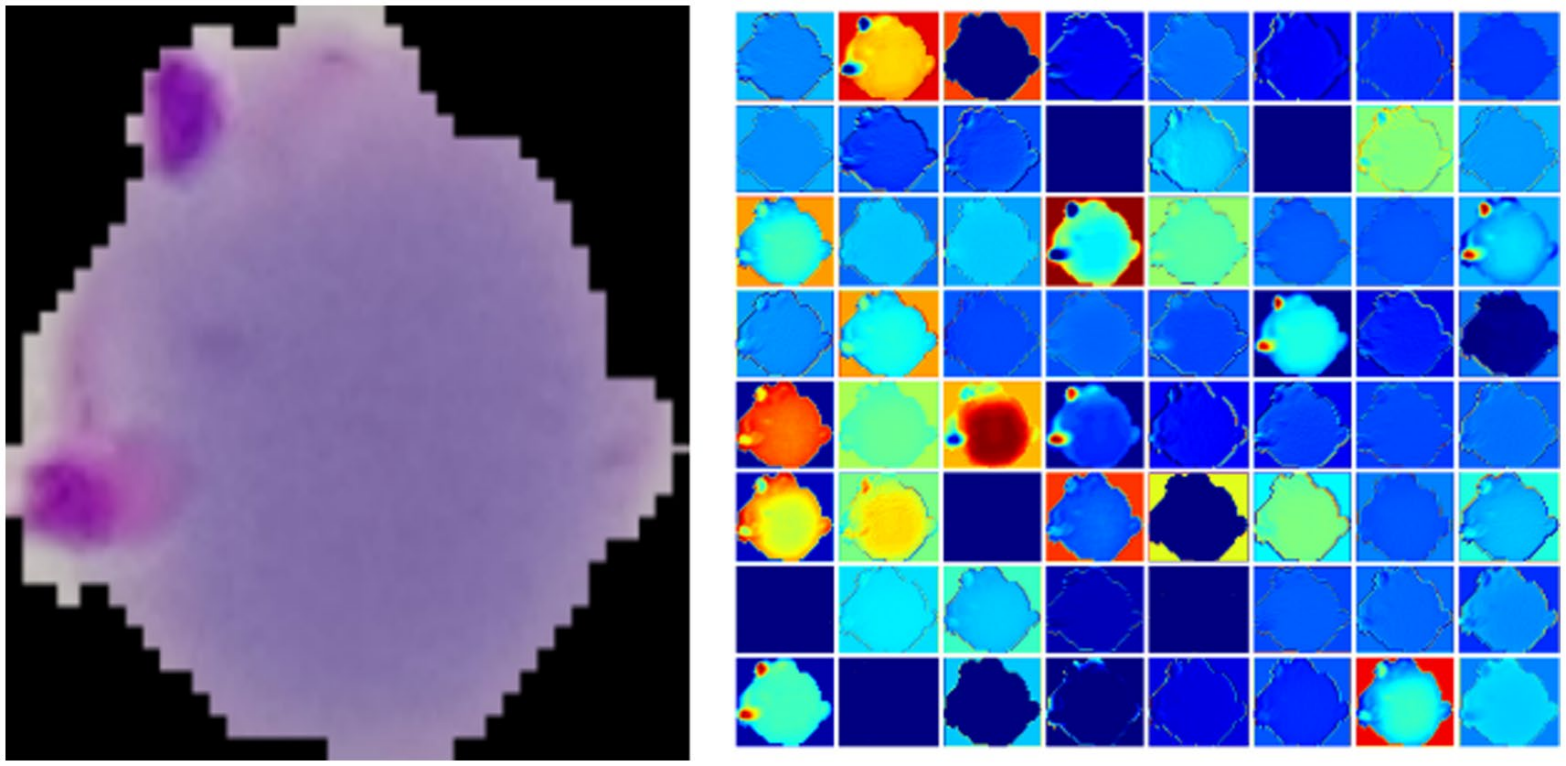
The left is a plasmodium image, and the right is a heat map.

**Figure 4:**
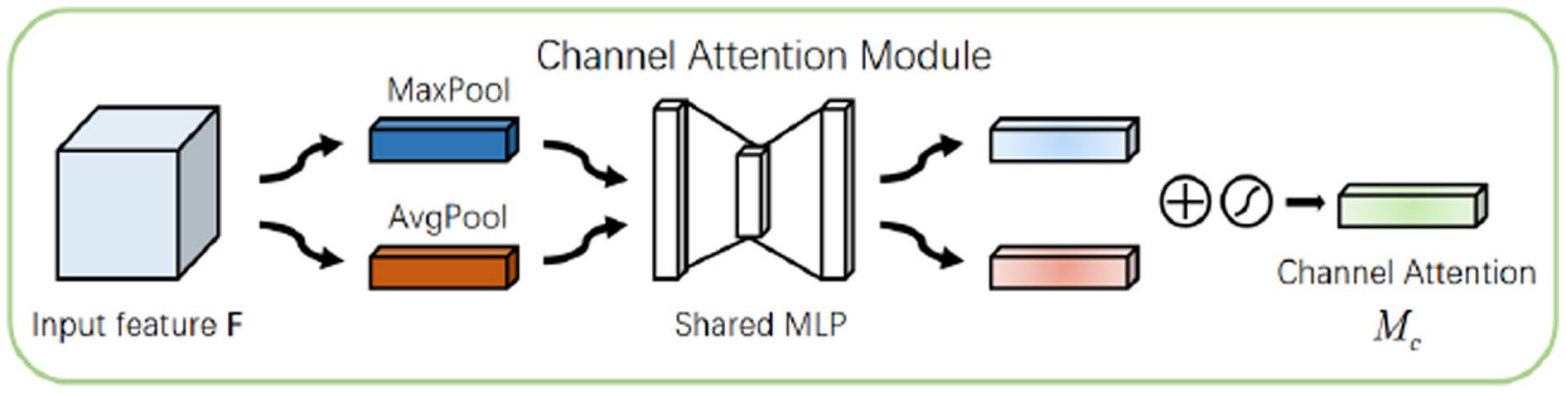
A channel attention block.

Figure 2 gives a plasmodium image and the corresponding heat map. From Figure 2, we find that there are noticeable differences between different channels (Figure 3). Thus, we can use the channel attention mechanism (13) to improve the classification accuracy of plasmodium. Figure 4 provides a channel attention module that can be formulated as


(1) $$\begin{array}{l} \mathrm{Mc}\left(F\right)=\sigma \left(\mathrm{MLP}\left(\mathrm{avgPoolO}\left(F\right)\right)\right)\\ \;\;\;\;\;\;\;\;\;\;\;\;\;\;\;\;\;+\sigma \left(\mathrm{MLP}\left(\mathrm{maxPool}\left(F\right)\right)\right) \end{array}$$

**Figure 5:**
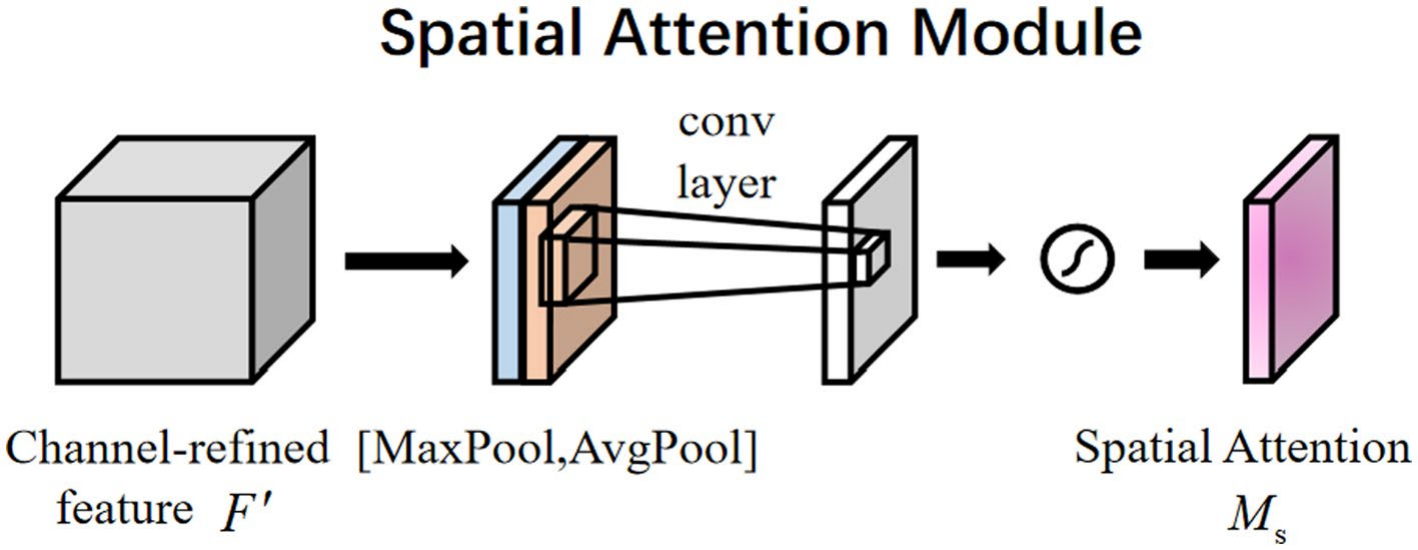
Spatial attention module.

The plasmodium randomly appears anywhere in the image. Therefore, the spatial attention mechanism (14) also applies to the classification of plasmodium. Different from channel attention, spatial attention focuses on ‘where’ the informative part lies, which is complementary to channel attention (15). To compute spatial attention, we first conducted average-pooling and max-pooling operations along the channel axis and concatenated them to generate an efficient feature descriptor. Data show that it is efficient to apply pooling operation along the channel axis in highlighting informative regions. Figure 5 displays a spatial attention module that can be described by


(2) $$\begin{array}{l} M_{s}\left(F\right)=\sigma \left(f^{7\times 7}\left(\left[\mathrm{AvgPool}\left(F\right)\mathrm{;MaxPool}\left(F\right)\right]\right)\right)\\ \;\;\;\;\;\;\;\;\;\;\;\;\;\;\;\;\;\;=\sigma \left(f^{7\times 7}\left(\left[F_{\mathrm{avg}}^{s}{\mathrm{;F}}_{\max}^{s}\right]\right)\right) \end{array}$$

## 4. Experiments

### 4.1. Training of the model

The training set was employed to construct the AI model, whereas the validation set was harnessed to assess the accuracy of classification performance of the established model.

**Figure 6:**
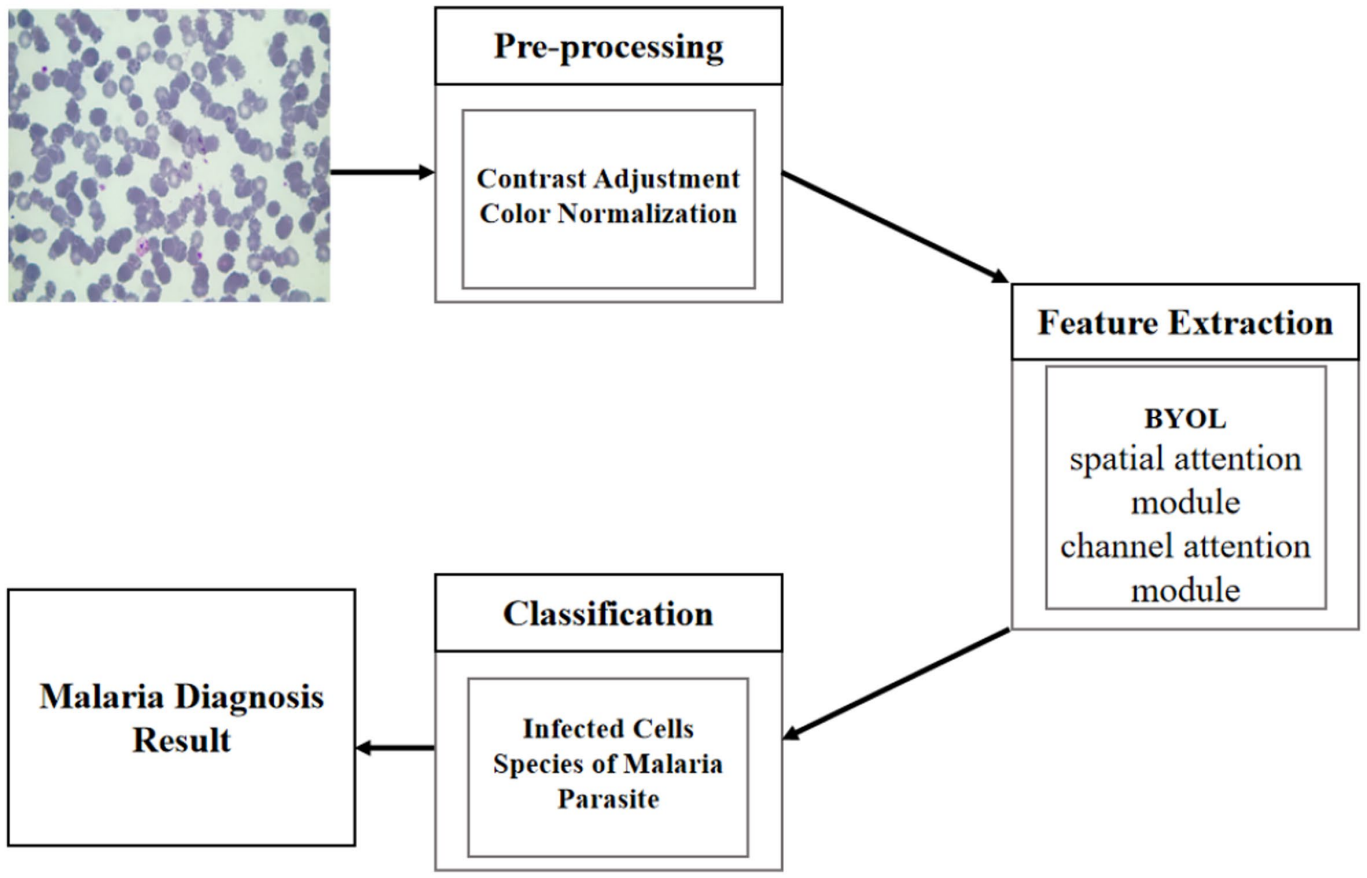
A schematic diagram of the algorithm.

With the application of the PyTorch platform, we adopted the ResNeSt-50 architecture pretrained and the BYOL to develop our AI algorithm. The retraining contained initializing the convolutional layers with loaded pretrained weights and updating the neural network to recognize plasmodium and non-plasmodium. The network structure was kept unchanged in this study. The weights of the last fully connected layer and the last three convolutional layers were tuned. After 50 epochs (iterations through the entire dataset), the training was terminated if no further improvements in accuracy or cross-entropy loss could be observed. The schematic diagram of our algorithm is exhibited in Figure 6.

### 4.2. The experimental results

**Figure 7:**
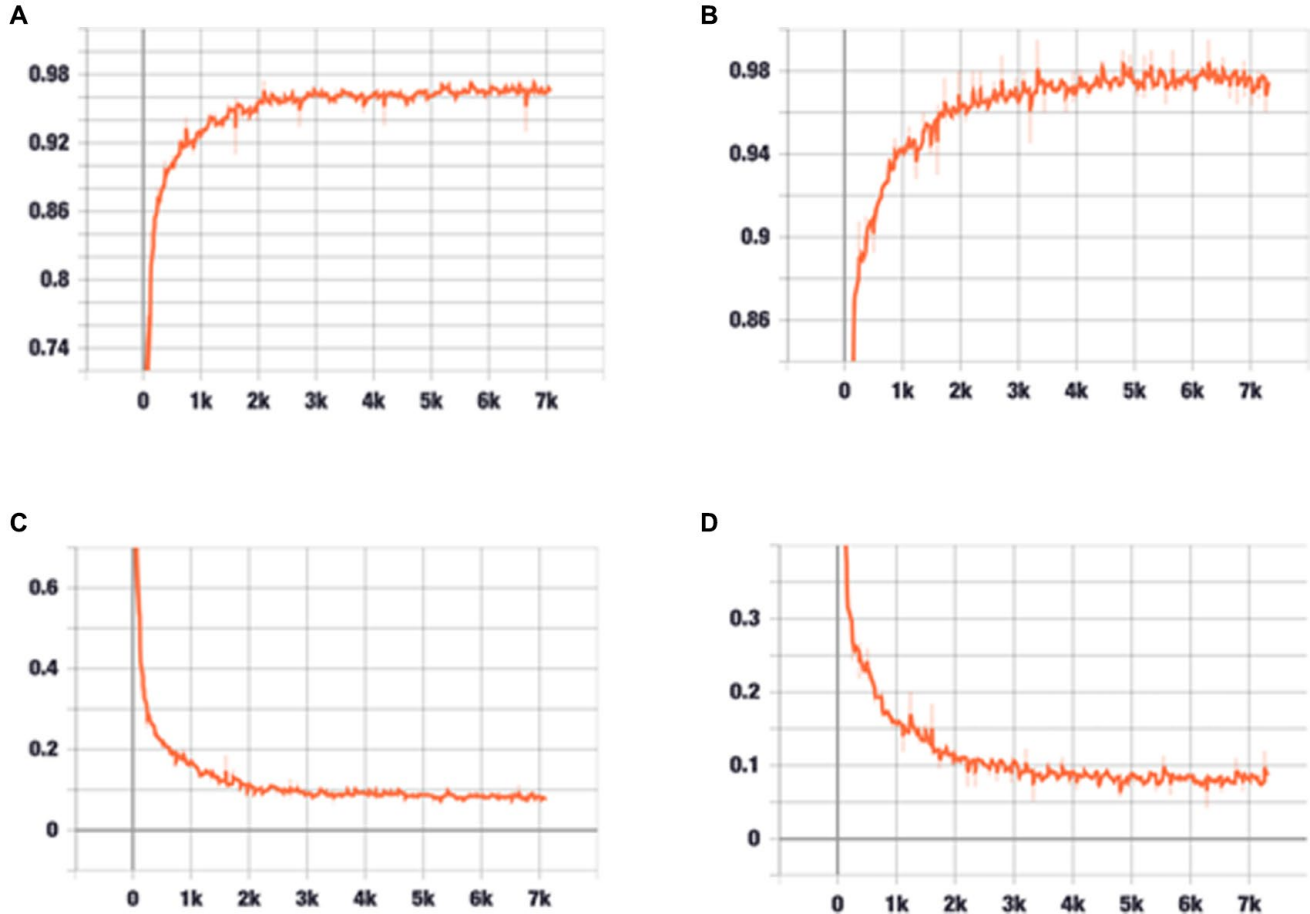
The performance of the model during training and validation. **(A)** Classification accuracy is plotted against training epochs. **(B)** The categorical cross-entropy loss is shown as a function of training epochs for the binary classification problem. **(C)** Classification accuracy is plotted against validation epochs. **(D)** The categorical cross-entropy loss is shown as a function of validation epochs for the binary classification problem. The graph shows that after about 1,000 rounds of training, the increase in accuracy rate slows down, and after about 2,000 rounds of training, the accuracy rate stabilizes at about 98%.

**Figure 8:**
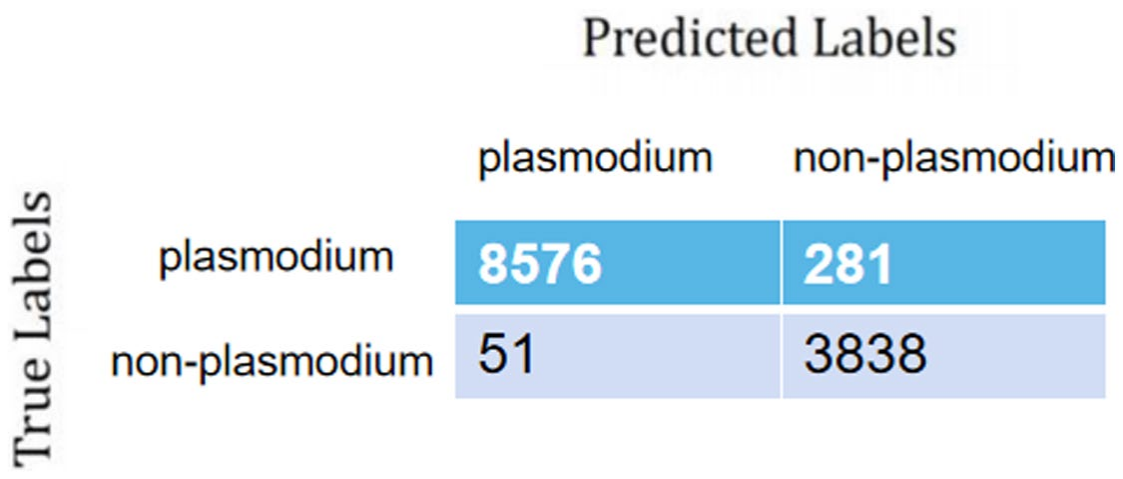
Confusion matrix of the AI framework.

During training and validation processes, accuracy and cross-entropy against the iteration step are plotted in Figure 7. Confusion matrix of the AI framework during the validation process is also displayed in Figure 8. The classification results of the test set are listed in Table 2. Given Figure 7 and Table 2, we can see that our training algorithm works and obtains the highest test accuracy of 97.8% compared to the other algorithms (Table 3).

**Table 2:** The diagnostic performances of the different algorithms on the test set.

Algorithms	Accuracy (%)	Sensitivity (%)	Specificity (%)
ResNeSt	85.7	84.7	87.7
ResNeSt+Image Preprocessing	87.2	86.6	88.3
ResNeSt+Image Preprocessing+BYOL	90.5	89.7	92.1
ResNeSt+ Image Preprocessing+BYOL+Spatial Attention	92.7	91.9	94.2
ResNeSt+ Image Preprocessing+BYOL+Spatial Attentino+Channel Attention	95.8	94.9	97.4
ResNeSt+ Image Preprocessing+BYOL+Spatial Attentino+Channel Attention	97.6	96.4	98.7
ResNeSt+ Image Preprocessing+OurBYOL+Spatial Attentino+Channel Attention	**97.8**	96.5	98.9

**Table 3:** A comparison between the AI algorithm and radiologist.

Object	Accuracy (%)
Physicians (*N* = 5)	90
AI modal	99

The experiment used the same dataset and all five physicians had more than 10 years of diagnostic experience.

## 5. Discussion

Malaria is a parasitic disease that seriously threatens human health. It primarily prevails in underdeveloped Africa, Southeast Asia, Oceania and Latin America where there is not enough capacity for detection. Detection is the key to the treatment of malaria cases and the control of infection sources. Microscopic examination and test paper are two commonly used detection methods with advantages such as rapidness, simplicity and low cost. The use of test paper is accompanied by the problems of false positive and false negative, and the antigen still exists in the blood for a long time even after the patient is cured. Therefore, microscopic examination is still regarded as the gold standard of malaria diagnosis. Notwithstanding, the outcome of microscopic examination largely depends on personal experience and skill levels.

## 6. Conclusion

The classification method of thin blood membranes of AI based on the self-supervision and attention mechanism is a training model founded on the whole pathological section, which can better enable clinicians to make decisions. The brightness, contrast, saturation, and hue of most Plasmodium images are in a similar range due to staining, so the channel attention mechanism is used to improve the feature extraction ability of the model, and the spatial attention mechanism is used for the feature that Plasmodium can appear in various areas of the images. By using the channel attention and spatial attention, the feature extraction ability of the model is enhanced; at the same time, the feature extraction ability of the model is also improved by using self-supervised learning to learn unlabeled data in response to the difficulty of image labeling and the difficulty of labeling. In terms of classification accuracy, it exceeds the average level of existing human doctors and paves the way for the future automatic technology of malaria detection.

## 7. Prospect

Our article has done a series of studies on Plasmodium classification and proposed a novel classification algorithm for Plasmodium, but there is a lack of research on the location detection and Plasmodium segmentation. So, we will conduct the following studies: 1. Subsequently, we will continue to study the image detection algorithm to get the position of Plasmodium on the picture to allow medical person to do better analysis. 2. Image segmentation of the Plasmodium picture to get the Plasmodium shape, so that the doctor can make the determination of the worm type.

Translated with www.DeepL.com/Translator (free version).

## Data availability statement

The data analyzed in this study is subject to the following licenses/restrictions: Contact first author for dataset use in deep learning. Requests to access these datasets should be directed to narcissist_fm@163.com.

## Ethics statement

The studies involving human participants were reviewed and approved by Ethics Committee of Wuhan Center for Disease Prevention and Control. The patients/participants provided their written informed consent to participate in this study.

## Author contributions

MF and KW: study concept and design. QZ and WH: acquisition of data. YL: image preprocessing. MF and LL: analysis and interpretation of data. MF and YL: drafting of the manuscript. LL and KW: critical revision of the manuscript for important intellectual content. All authors contributed to the article and approved the submitted version.

## Funding

This study was supported by the Foundation for General Project of Health Commission of Hubei Province from 2021 to 2022 (Grant Number: WJ2021M024).

## Conflict of interest

The authors declare that the research was conducted in the absence of any commercial or financial relationships that could be construed as a potential conflict of interest.

## Publisher’s note

All claims expressed in this article are solely those of the authors and do not necessarily represent those of their affiliated organizations, or those of the publisher, the editors and the reviewers. Any product that may be evaluated in this article, or claim that may be made by its manufacturer, is not guaranteed or endorsed by the publisher.
